# Administration of Oxygen Ultra-Fine Bubbles Improves Nerve Dysfunction in a Rat Sciatic Nerve Crush Injury Model

**DOI:** 10.3390/ijms19051395

**Published:** 2018-05-07

**Authors:** Hozo Matsuoka, Kosuke Ebina, Hiroyuki Tanaka, Makoto Hirao, Toru Iwahashi, Takaaki Noguchi, Koji Suzuki, Shunsuke Nishimoto, Tsuyoshi Murase, Hideki Yoshikawa

**Affiliations:** 1 Department of Orthopaedic Surgery, Osaka University Graduate School of Medicine, 2-2 Yamadaoka, Suita 565-0871, Osaka, Japan; go_go_475_going_my_way@yahoo.co.jp (H.M.); tanahiro-osk@umin.ac.jp (H.T.); makohira777@gmail.com (M.H.); kurobuchi0918@gmail.com (T.I.); tmurase-osk@umin.ac.jp (T.M.); yhideki@ort.med.osaka-u.ac.jp (H.Y.); 2 Department of Orthopaedic Surgery, National Hospital Organization, Osaka Minami Medical Center, 2-1 Kidohigashi, Kawachinagano 586-8521, Osaka, Japan; n-takaaki@hotmail.co.jp; 3 Department of Orthopaedic Surgery, Kansai Rosai Hospital, 3-1-69, Inabaso, Amagasaki 660-0064, Hyogo, Japan; kouji_szk@hotmail.co.jp (K.S.); tennishun37@gmail.com (S.N.)

**Keywords:** ultra-fine bubbles, oxygen ultra-fine bubbles, sciatic nerve crush injury, dorsal root ganglion neurons, neurite outgrowth, Schwann cells, proliferation, peripheral nerve, regeneration

## Abstract

Ultra-fine bubbles (<200 nm in diameter) have several unique properties and have been tested in various medical fields. The purpose of this study was to investigate the effects of oxygen ultra-fine bubbles (OUBs) on a sciatic nerve crush injury (SNC) model rats. Rats were intraperitoneally injected with 1.5 mL saline, OUBs diluted in saline, or nitrogen ultra-fine bubbles (NUBs) diluted in saline three times per week for 4 weeks in four groups: (1) control, (sham operation + saline); (2) SNC, (crush + saline); (3) SNC+OUB, (crush + OUB-saline); (4) SNC+NUB, (crush + NUB-saline). The effects of the OUBs on dorsal root ganglion (DRG) neurons and Schwann cells (SCs) were examined by serial dilution of OUB medium in vitro. Sciatic functional index, paw withdrawal thresholds, nerve conduction velocity, and myelinated axons were significantly decreased in the SNC group compared to the control group; these parameters were significantly improved in the SNC+OUB group, although NUB treatment did not affect these parameters. In vitro, OUBs significantly promoted neurite outgrowth in DRG neurons by activating AKT signaling and SC proliferation by activating ERK1/2 and JNK/c-JUN signaling. OUBs may improve nerve dysfunction in SNC rats by promoting neurite outgrowth in DRG neurons and SC proliferation.

## 1. Introduction

Ultra-fine bubbles (UFBs), also referred to as nanobubbles, are miniature gas bubbles <200 nm in diameter in liquids that possess many unique physical properties [1,2]. UFBs remain stable in liquids for long periods at a high concentration owing to their negatively charged surface and high internal pressure, whereas macrobubbles (>50 μm in diameter), increase in size and rapidly burst at the surface of liquids [3,4]. Previous reports have demonstrated that UFBs increase the oxygen pressure in liquids to a greater extent than microbubbles (10–50 μm in diameter) [1,4], and the high oxygen gas solubility of UFBs can be beneficial for oxygenation of hypoxic tissues [5,6,7]. Nano-technologies are gaining recognition in the medical field, and their usefulness for ultrasound imaging and drug delivery has been demonstrated [8]. We previously demonstrated that administration of oxygen ultra-fine bubbles (OUBs) and air ultra-fine bubbles promotes the growth of plants, animals, and fish [2], and that OUBs prevent bone loss in glucocorticoid-induced osteoporosis in mice by suppressing osteoclast differentiation [9]. Moreover, oral administration of OUBs in water reduces calcium oxalate deposits in rat kidney [10], indicating its usefulness for clinical applications. However, no reports have assessed the effects of OUBs on the nervous system.

Peripheral nerve injury (PNI) is usually caused by accidental trauma, metabolic or inherited genetic diseases, or surgical procedures, and may lead to permanent denervation, movement limitations, sensory disruptions, and neuropathic pain [11,12]. Currently, repair of the peripheral nervous system (PNS) is still a major problem in clinical treatments, and nerve regeneration has been the focus of research in basic and clinical medicine in the field of neuroscience. Unlike the central nervous system, the PNS has a far greater capacity to regenerate towards its original target and recover functionally in response to injuries. However, regeneration after PNI is not always complete, especially in elderly patients and in the case of chronic denervation and injuries to large peripheral nerve trunks, such as the brachial and lumbar plexus [13,14,15]. Moreover, injured nerves may have impaired oxygenation due to a compromised blood supply and edema that trigger a vicious cycle of further hypoxia [16].

Favorable effects of hyperbaric oxygenation have been reported for healing of mechanically damaged peripheral nerves induced by nerve transection or crushing injury, both in animal models [17,18] and humans [19,20]. However, this treatment has some limitations, including the high cost of hyperbaric chamber treatment [21]. Therefore, to overcome these drawbacks, we hypothesized that OUB administration may improve hypoxic conditions at the injured site and may have beneficial effects on nerve regeneration.

The main constituents of the PNS are neurons and Schwann cells (SCs), and after PNI, the isolated distal axons undergo Wallerian degeneration. SCs at the injury site de-differentiate, proliferate, and line the endoneurial tubes to guide regenerating axons. Finally, SCs redifferentiate and remyelinate after guiding axons toward the target [22]. Thus, not only neurons but also SCs play a very important role in peripheral nerve regeneration. In this study, we assessed the effects of intraperitoneal administration of OUBs in a rat model of sciatic nerve crush injury (SNC) and the influence of OUBs on isolated dorsal root ganglion (DRG) neurons and SCs in vitro.

## 2. Results

### 2.1. OUBs Improve Dysfunction after Sciatic Nerve Crush Injury in Rats

We assessed the effects of OUB administration in a SNC rat model, which was established as previously described [23]. The rats were given 1.5 mL saline, OUBs diluted in saline, or nitrogen ultra-fine bubbles (NUBs) diluted in saline by intraperitoneal injection three times per week for 4 weeks on the day after crush or sham operation. Rats were divided into four groups: (1) control, sham operation + saline injections; (2) SNC, crush injury + saline injections; (3) SNC+OUB, crush injury + OUB-saline injections; and (4) SNC+NUB, crush injury + NUB-saline injections.

**Figure 1:**
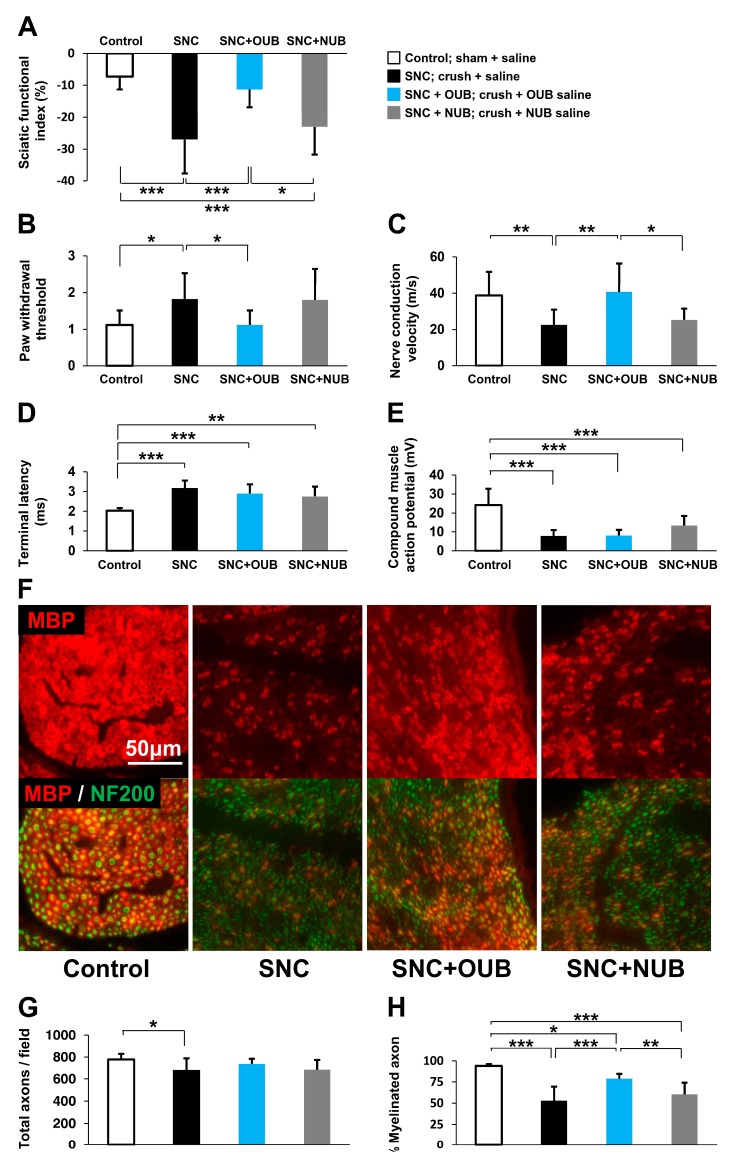
Oxygen ultra-fine bubbles (OUBs) improve dysfunction after sciatic nerve crush injury in rats. (**A**–**E**) Sciatic functional index analysis (**A**), von Frey filament test (**B**), and electrophysiological analysis (**C**); nerve conduction velocity (**D**); terminal latency (**E**); compound muscle action potential were performed 4 weeks after sciatic nerve crush injury in each group (Control, SNC, SNC+OUB, SNC+NUB); (**F**) representative fluorescence micrographs of cross-sectional slices of sciatic nerves labeled for MBP (red) and NF200 (green) 4 weeks after operation. Scale bar = 50 μm; (**G**) quantification of total axons (NF200-positive axons) per field; (**H**) quantification of myelinated axons (MBP-positive axons) per total axons (NF200-positive axons). Significance was determined by one-way ANOVA followed by Tukey-Kramer HSD test. Graphs show mean ± SD. (*n* = 11 for the control group, *n* = 15 for the SNC group, *n* = 11 for the SNC+OUB group, and *n* = 8 for the SNC+NUB group). * *p* < 0.05, ** *p* < 0.01, *** *p* < 0.001. OUBs: oxygen ultra-fine bubbles, SNC: sciatic nerve crush injury, NUB: nitrogen ultra-fine bubble, MBP: myelin basic protein, NF200: neurofilament 200.

Motor recovery after SNC was assessed by sciatic functional index (SFI) analysis. The SFI was significantly decreased in SNC rats compared to control rats (SFI (%): control vs. SNC: −7.3 vs. −27.0; *p* < 0.001). OUB treatment prevented these reductions in the SFI (SNC vs. SNC+OUB: −27.0 vs. −11.3; *p* < 0.001), although NUB treatment had no significant effects compared to the SNC group (Figure 1A).

Next, sensory function was measured with the von Frey filament test. Paw withdrawal thresholds were significantly higher in SNC rats than control rats (control vs. SNC: 1.1 vs. 1.8; *p* < 0.05), whereas OUB treatment improved this deterioration of the mechanical thresholds (SNC vs. SNC+OUB: 1.8 vs. 1.1; *p* < 0.05), but NUB treatment showed no significant effects compared to the SNC group (Figure 1B).

Electrophysiological analysis revealed that nerve conduction velocity (NCV) was significantly decreased in SNC rats compared to control rats (control vs. SNC: 38.8 vs. 22.5 m/s; *p* < 0.01), whereas OUB treatment (SNC vs. SNC+OUB: 22.5 vs. 40.7 m/s; *p* < 0.01), but not NUB treatment, led to significant recovery (Figure 1C). On the other hand, terminal latency (TL) and compound muscle action potential (CMAP) were significantly deteriorated in the SNC group compared to the control group (TL: control vs. SNC: 2.0 vs. 3.2 ms; *p* < 0.001; CMAP: control vs. SNC: 24.2 vs. 7.8 mV; *p* < 0.001), and these parameters were not significantly restored by either OUB (TL: SNC vs. SNC+OUB: 3.2 vs. 2.9 ms; *p* = 0.26; CMAP: SNC vs. SNC+OUB: 7.8 vs. 8.0 mV; *p* = 0.99) or NUB (TL: SNC vs. SNC+NUB: 3.2 vs. 2.8 ms; *p* = 0.07; CMAP: SNC vs. SNC+NUB: 7.8 vs. 13.4 mV; *p* = 0.08) treatment (Figure 1D,E).

To investigate remyelination by SCs, histological analysis of the sciatic nerve was performed 4 weeks after the operation (Figure 1F). The number of axons was significantly decreased in SNC rats compared to control rats (control vs. SNC: 778 vs. 677 axons/field; *p* < 0.05). OUB but not NUB treatment showed a tendency to increase the number of axons, although did not reach statistical significance (SNC vs. SNC+OUB: 677 vs. 736 axons/field; *p* = 0.28; SNC vs. SNC+NUB: 677 vs. 683 axons/field; *p* = 0.99). (Figure 1G). The ratio of myelinated axons was significantly decreased in SNC rats compared to control rats (control vs. SNC: 93.9 vs. 53.0%; *p* < 0.001), whereas OUB treatment produced significant recovery (SNC vs. SNC+OUB: 53.0 vs. 78.7%; *p* < 0.001), although this recovery was incomplete compared with control rats (control vs. SNC+OUB: 93.9 vs. 78.7%; *p* < 0.05). NUB treatment showed no significant effects compared to SNC rats (Figure 1H). These findings demonstrate that OUBs promote nerve regeneration and functional recovery in a rat SNC model.

### 2.2. OUBs Promote Neurite Outgrowth in DRG Neurons

**Figure 2:**
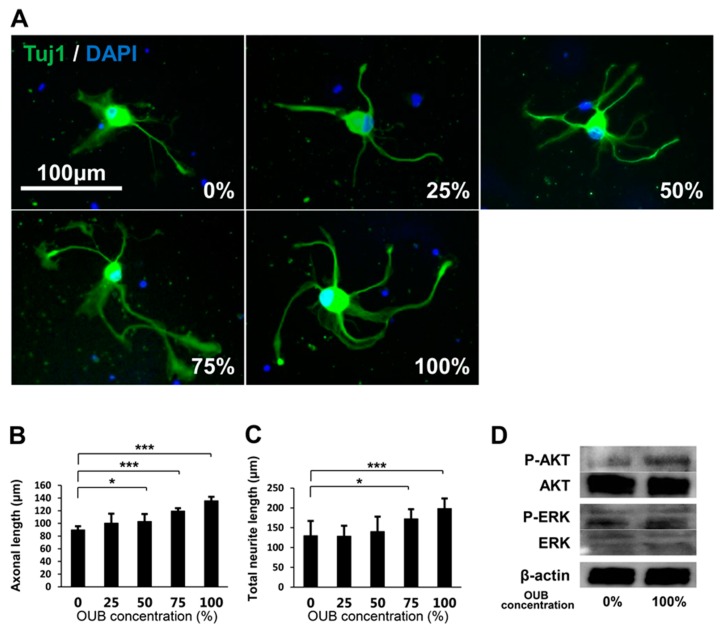
OUBs promote neurite outgrowth in DRG neurons. (**A**) Representative fluorescence micrographs of DRG neurons cultured for 48 h in OUBs diluted in Sato medium (concentration 0, 25, 50, 75, and 100%). Cells were co-stained with Tuj1 (green) together with DAPI (blue). Scale bar = 100 μm. (**B**,**C**) Quantification of the axonal length (the length of the longest neurite per TuJ1-positive neuron) (**B**) and total neurite length per neuron (**C**). The axonal length and total neurite length were calculated from at least 30 neurons in each experiment; (**D**) western blotting of the neurite outgrowth related signals in cultured DRG neurons with or without OUBs. β-actin was used as an internal control. The data were analyzed by one-way ANOVA followed by Dunnett test. Graphs show mean ± SD. Results are representative of eight independent experiments. * *p* < 0.05, *** *p* < 0.001 compared with 0% group. OUBs: oxygen ultra-fine bubbles, DRG: dorsal root ganglion, Tuj1: neuronal class III β-tubulin, ERK: extracellular signal-regulated kinase.

The effects of OUBs on neurite outgrowth in DRG neurons were evaluated with OUBs diluted in Sato medium (concentration 0, 25, 50, 75, and 100%) (Figure 2A). OUBs increased axonal length and total neurite length in a dose-dependent manner, and significant differences compared to control (0%) were observed at concentrations ≥50% and 75% OUBs (Figure 2B,C). We investigated cell signaling with western blotting (WB), which revealed that OUBs upregulated phosphorylation of AKT, whereas OUBs did not affect phosphorylation of extracellular signal-regulated kinase1/2 (ERK1/2) (Figure 2D). These findings suggest that AKT signaling is involved in OUB-induced neurite outgrowth activity of DRG neurons.

### 2.3. OUBs Have No Effects on the Differentiation of SCs

**Figure 3:**
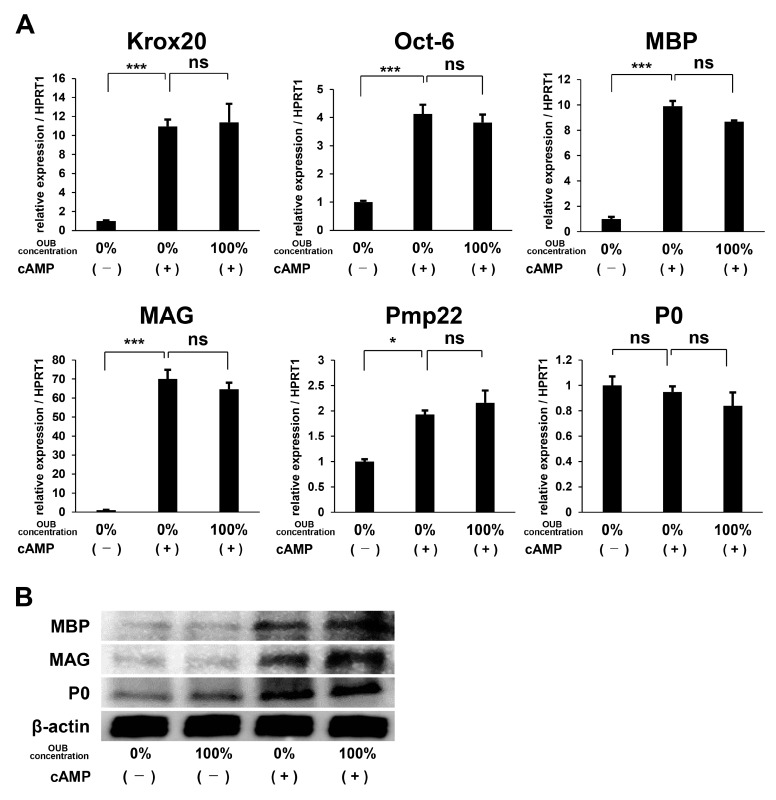
OUBs have no effects on the differentiation of Schwann cells (SCs). (**A**) Change in gene expression for differentiation makers including myelination-associated transcription factors (Krox20 and Oct-6) and myelin genes (MBP, MAG, Pmp22, and P0) were examined using SCs cultured in the differentiation medium containing db-cAMP with or without OUBs. HPRT1 was used as an internal control; (**B**) western blotting of myelin-related proteins (MBP, MAG, and P0) in SCs cultured in both growth medium and differentiation medium containing db-cAMP with or without OUBs. β-actin was used as an internal control. The data were analyzed by one-way ANOVA followed by Dunnett test. Graphs show indicate mean ± SD. Results are representative of three independent experiments. * *p* < 0.05, *** *p* < 0.001 compared with 0% in differentiation medium. ns: not significant. OUBs: oxygen ultra-fine bubbles, SCs: Schwann cells, Oct-6: octamer transcription factor-6, MBP: myelin basic protein, MAG: myelin-associated glycoprotein, Pmp22: peripheral myelin protein 22, P0: protein zero, and HPRT1: hypoxanthine phosphoribosyltransferase 1.

To evaluate the effects of OUBs on SC differentiation, we focused on the expression of differentiation markers including myelination-associated transcription factors such as Krox20 and octamer transcription factor-6 (Oct-6) and myelin-related proteins such as myelin basic protein (MBP), myelin-associated glycoprotein (MAG), peripheral myelin protein 22 (Pmp22), and protein zero (P0). SCs were incubated in growth medium or differentiation medium containing dibutyryl cyclic AMP (db-cAMP) with or without OUBs. Real-time polymerase chain reaction (PCR) analysis showed that OUBs did not significantly affect expression of differentiation-related mRNAs (Krox20, Oct-6, MBP, MAG, Pmp22, and P0) in the differentiation medium (Figure 3A). In addition, WB revealed that OUBs did not stimulate the expression of myelin-related proteins (MBP, MAG, and P0) in either growth medium or differentiation medium, although the expression of each protein in differentiation medium increased compared with the expression in the growth medium (Figure 3B). Our data indicate that OUBs do not affect SC differentiation either positively or negatively.

### 2.4. OUBs Stimulate the Proliferation of SCs

**Figure 4:**
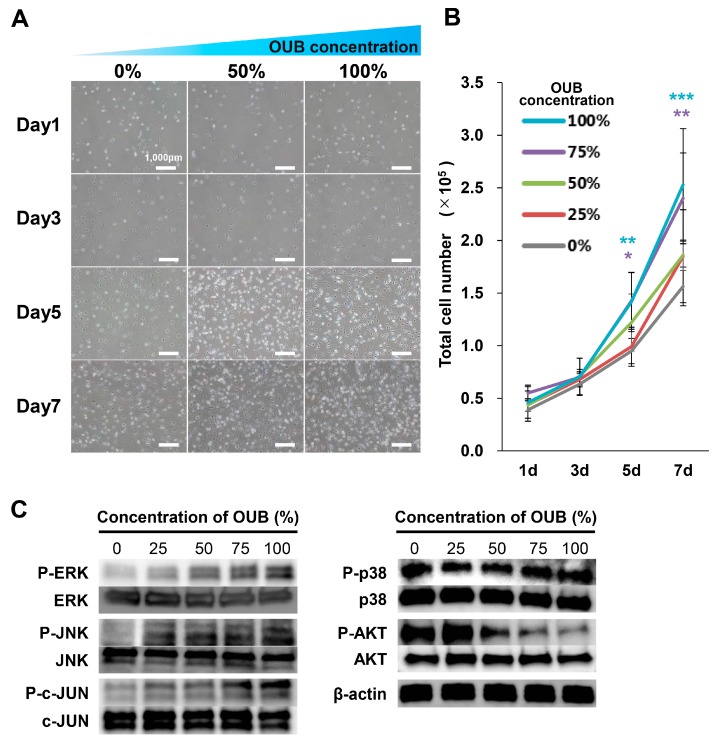
OUBs stimulate the proliferation of SCs. (**A**) Representative micrographs of SCs cultured at 1, 3, 5, and 7 days in OUBs diluted in growth medium (concentration 0, 50, and 100%). Scale bar = 1000 μm; (**B**) quantification of the total cell number of SCs in OUBs diluted in growth medium (concentration 0, 25, 50, 75, and 100%); (**C**) western blotting of the proliferation and differentiation related signals in SCs cultured in OUBs diluted in growth medium for 30 min (concentration 0, 25, 50, 75, and 100%). β-actin was used as an internal control. The data were analyzed by one-way ANOVA followed by Dunnett test. Graphs show mean ± SD. Results are representative of more than four independent experiments. * *p* < 0.05, ** *p* < 0.01, *** *p* < 0.001 compared with 0% group. OUBs: oxygen ultra-fine bubbles, SCs: Schwann cells, ERK: extracellular signal-regulated kinase, JNK: c-Jun-N-terminal kinase.

The effects of OUBs on SC proliferation were evaluated with a cell proliferation assay. SCs were cultured in growth medium containing OUBs (concentration 0, 25, 50, 75, and 100%) for 1, 3, 5, and 7 days, and the total cell number was then counted. Figure 4A shows representative micrographs of SCs cultured in OUBs diluted in growth medium (concentration 0, 50, and 100%). The growth curve showed that OUBs increased the total cell number in a dose-dependent manner, and a significant difference compared to control (0%) was observed at more than 75% OUBs on days 5 and 7 (Figure 4B). Regarding cell signaling, WB revealed that OUBs upregulated the phosphorylation of ERK1/2 and c-Jun-N-terminal kinase (JNK)/c-JUN in a dose-dependent manner, whereas OUBs downregulated phosphorylation of AKT (Figure 4C). These findings indicate that ERK1/2 and JNK/c-JUN signaling is involved in the proliferation-promoting activity of OUBs in SCs.

### 2.5. OUBs Induce the Expression of Regeneration-Related Factors in SCs

**Figure 5:**
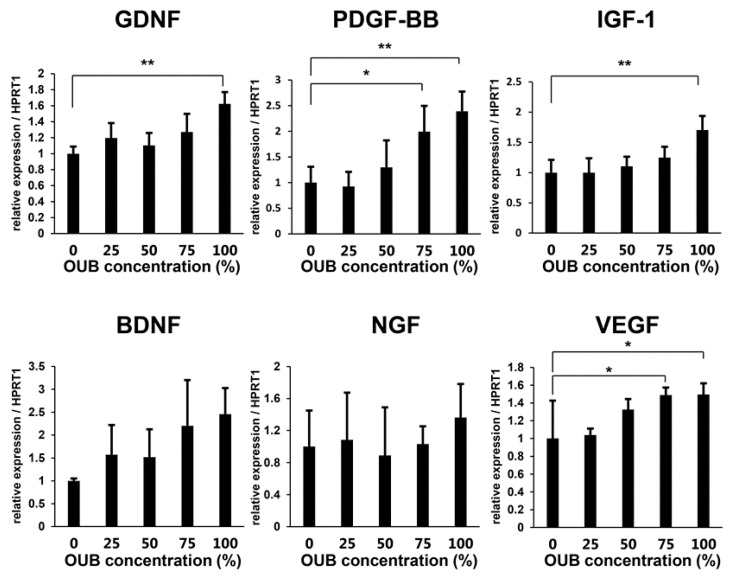
OUBs induce the expression of regeneration-related factors in SCs. Change in gene expression for regeneration-related factors (GDNF, PDGF-BB, IGF-1, BDNF, NGF, and VEGF) were examined using SCs cultured in OUBs diluted in medium (concentration 0, 25, 50, 75, and 100%). HPRT1 was used as an internal control. The data were analyzed by one-way ANOVA followed by a Dunnett test. Graphs show mean ± SD. Results are representative of three independent experiments. * *p* < 0.05, ** *p* < 0.01 compared with 0% group. OUBs: oxygen ultra-fine bubbles, SCs: Schwann cells, GDNF: glial cell-derived neurotrophic factor, PDGF-BB: platelet-derived growth factor-beta, IGF-1: insulin-like growth factor-1, BDNF: brain-derived neurotrophic factor, NGF: nerve growth factor, VEGF: vascular endothelial growth factor, HPRT1: hypoxanthine phosphoribosyltransferase 1.

Induction of neurotrophic factors such as glial cell-derived neurotrophic factor (GDNF) and brain-derived neurotrophic factor (BDNF) in SCs is essential for neuronal survival and peripheral nerve regeneration, and this function of SCs appears to be principally driven by c-JUN [24]. The upregulation of phosphorylated JNK/c-JUN signaling, described above, led us to hypothesize that OUBs would induce SCs to express neurotrophic factors to promote nerve regeneration. Thus, the expression of various trophic factors from SCs in growth medium with or without OUBs was evaluated. Real-time PCR analysis revealed that OUBs significantly promoted the mRNA expression of regeneration-related factors such as GDNF, platelet-derived growth factor-beta (PDGF-BB), insulin-like growth factor-1 (IGF-1), and vascular endothelial growth factor (VEGF) in a dose-dependent manner. The same tendency was observed for mRNA expression of BDNF and nerve growth factor (NGF), but the differences were not significant (Figure 5). These findings suggest that OUBs induce the expression of regeneration-related factors in SCs, which may contribute to nerve regeneration.

### 2.6. OUBs Accelerate Proliferation and Inhibit Apoptosis of SCs under Hypoxic Conditions In Vitro

**Figure 6:**
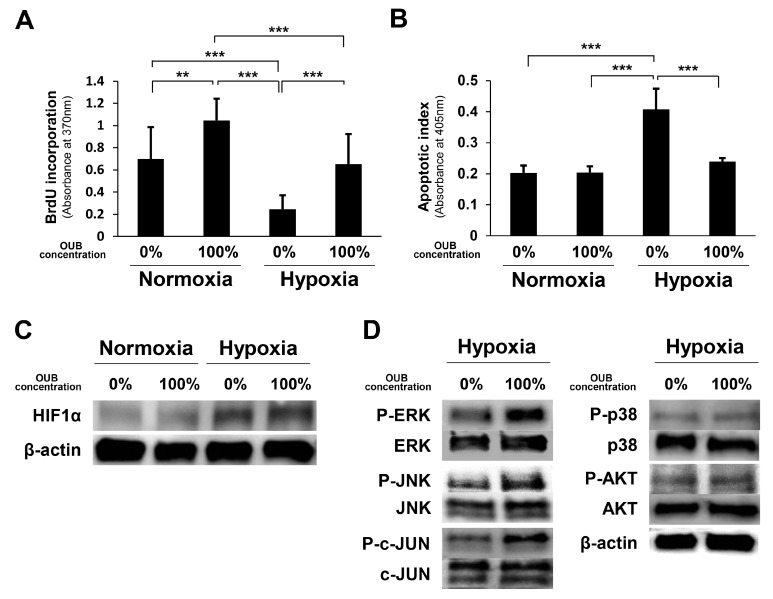
OUBs accelerate proliferation and inhibit apoptosis of SCs under hypoxic conditions in vitro. SCs were cultured in growth medium with or without OUBs under both normoxic conditions (20% O_2_) and hypoxic conditions (1% O_2_). (**A**) The proliferation rate of SCs was detected by the BrdU uptake assay; (**B**) hypoxic induced cell death was evaluated by an apoptosis assay; (**C**) western blotting of HIF1α in SCs under the same conditions. β-actin was used as an internal control; (**D**) western blotting of the proliferation and differentiation related signals in SCs cultured in growth medium with or without OUBs under hypoxic conditions. The data were analyzed by one-way ANOVA followed by Tukey-Kramer HSD test. Graphs show mean ± SD. Results are representative of more than five independent experiments. ** *p* < 0.01, *** *p* < 0.001. OUBs: oxygen ultra-fine bubbles, SCs: Schwann cells, BrdU: 5-bromo-2-deoxyuridine, HIF1α: hypoxia-inducible factor 1α, ERK: extracellular signal-regulated kinase, JNK: c-Jun-N-terminal kinase.

As the oxygen level affects the viability of SCs [21], and persistent hypoxia (partial pressure of oxygen <10 mmHg) is observed at the injury site after PNI [25], the effects of OUBs on SC proliferation and apoptosis were evaluated under both normoxic conditions (20% O_2_) and hypoxic conditions (1% O_2_). The 5-bromo-2-deoxyuridine (BrdU) uptake assay revealed that OUBs significantly accelerated the proliferation rate compared to control (0%) under normoxic conditions, and the BrdU incorporation rate was significantly decreased in non-OUBs (0%) under hypoxic conditions, compared to the control (0%) under normoxic conditions (20% O_2_); OUB treatment under hypoxic conditions resulted in significant recovery (Figure 6A). An apoptosis assay revealed that apoptotic activity was significantly increased in non-OUBs (0%) under hypoxic conditions compared with control (0%) and 100% OUBs under normoxic conditions, whereas OUB treatment significantly improved this deterioration under hypoxic conditions (Figure 6B). WB revealed that protein levels of hypoxia-inducible factor 1α (HIF1α) were promoted by hypoxia compared to normoxia, although no apparent difference was observed between non-OUB- (0%) and 100% OUB-treated SCs (Figure 6C). Regarding cell signaling, WB revealed that OUBs upregulated the phosphorylation of ERK1/2 and JNK/c-JUN compared with non-OUBs (0%), even under hypoxic conditions (Figure 6D). These findings indicate that OUBs also promote the proliferation of SCs via HIF1α-independent, ERK1/2 and JNK/c-JUN signaling pathways, even under hypoxic conditions.

## 3. Discussion

To the best of our knowledge, this is the first report demonstrating the effect of OUBs on nerve regeneration. Our results revealed that OUBs promoted nerve regeneration and functional recovery in SNC rats.

Previous studies showed that impaired oxygenation due to a compromised blood supply induces persistent hypoxia at the injury site after PNI, and an insufficient supply of oxygen may result in the death of migratory SCs and loss of function of newly regenerated axons [16,17,21].

Oxygen microbubbles (<50 µm in diameter) improve hypoxic conditions in blood in a dose-dependent manner, suggesting that oxygen microbubbles may be a potentially effective tool for the oxygenation of hypoxic tissue [4]. In addition, clinical applications of microbubbles and nanobubbles have been reported. Drugs encapsulated in microbubbles can be focally released at a target tissue and incorporated by various cells [26,27,28,29,30]. Oral intake of OUB-containing water reduces the expression of monocyte chemoattractant protein-1 in the kidneys of ethylene glycol-treated rats [10]. Moreover, we previously demonstrated that intraperitoneal administration of OUBs increases the number of serum nanoparticles in mice [9]. Taken together, OUBs may affect target cells in vivo and in vitro, and we hypothesized that OUB administration may be effective in improving hypoxic conditions and ameliorating dysfunction in SNC rats.

Regarding signaling pathways in neurons, AKT and ERK signaling molecules are profoundly implicated in the regulation of axonal outgrowth and branching in sensory neurons [31,32], and both types of signaling are important for nerve development and regeneration [33]. In this study, OUBs upregulated phosphorylation of AKT in DRG neurons, although ERK1/2 phosphorylation was unaffected. Previous studies have demonstrated cross-talk between AKT and ERK signaling pathways [34,35]. Furthermore, AKT inhibits ERK signaling in a tissue-specific manner [36,37]. Therefore, the deficit in ERK activation may be a consequence of an increase in either phosphorylated AKT, or another, as yet unidentified, signaling pathway.

In SCs, mitogen-activated protein kinases (e.g., ERK, p38, and JNK) are rapidly and highly activated and play crucial roles in nerve regeneration after injury [38,39,40,41]. SCs can de-differentiate and proliferate in response to nerve injury via activation of ERK1/2 as part of Wallerian degeneration, and c-JUN activation by JNK is also essential for SC migration and proliferation [42,43]. On the other hand, AKT phosphorylation plays an important role in SC differentiation and myelination [39]. In our study, OUBs upregulated phosphorylation of ERK, JNK, and c-JUN, although OUBs downregulated phosphorylation of AKT in SCs. However, the expression of myelin-related proteins in SCs was not affected by OUBs, despite the downregulation of phosphorylated AKT. The expression of myelin-related proteins reached a peak at 36–48 h [44,45], whereas, we evaluated expression of those proteins in SCs after 72 h. This difference in the time point of evaluation may have affected our results. Taken together, OUBs may promote neurite outgrowth in DRG neurons via AKT signaling and SC proliferation via ERK1/2 and JNK/c-JUN signaling during nerve regeneration, which may lead to the increased ratio of myelinated axons and result in the recovery of motor and sensory function and NCV.

SCs release regeneration-related factors such as GDNF, PDGF-BB, IGF-1, BDNF, NGF, and VEGF [46,47,48,49,50], which are essential for the promotion of axonal outgrowth [51]. VEGF is also capable of stimulating vascularization and angiogenesis, which promote the restoration of the oxygen supply to regenerating nerve tissues [52]. SCs express GDNF and BDNF via JNK/c-JUN signaling [24,53]. Taken together, OUBs may provide a favorable milieu for axonal regeneration by promoting expression of GDNF, PDGF-BB, IGF-1, and VEGF by SCs, possibly via JNK/c-JUN signaling.

Regarding electrophysiological analysis, a previous report showed that NCV is correlated with myelin thickness and internode distance [54]. In our study, OUBs promoted the recovery of NCV, presumably due to the recovery of the ratio of myelinated axons. TL includes the conduction time from the stimulating site to the distal end and also the transmission time across the neuromuscular junction [55]. CMAP is a total indicator from the nerve injury site to the target organ, whereas NCV is a local indicator of nerve regeneration near the injury site [23]. These distinctions may explain the difference between the recovered NCV and the unrecovered TL and CMAP following OUB treatment. These results suggest that OUBs promote nerve regeneration around the injury site, but that recovery around the neuromuscular junction is premature 4 weeks after the injury.

In this study, OUBs did not affect the expression of HIF1α under hypoxic conditions, similar to results we previously reported in osteoclasts and osteoblasts in mice [9]. The precise mechanisms of how OUBs affect cell signaling (receptor, endocytosis, etc.) remain to be elucidated.

Our study has some limitations. First, although we previously demonstrated that intraperitoneal administration of OUBs increases the number of serum nanoparticles in mice [9], the size of UFBs is so small that detection of the bioavailability of UFBs in focal tissues is technically difficult. Second, DRG neurons and SCs may respond differently to OUBs. Third, our preliminary experiments, using a high concentration of NUBs diluted in medium, showed some apoptosis of cultured cells, which was never seen in OUB experiments, and thus, evaluation of the effects of NUBs in vitro was difficult. Taken together, the effects of OUBs on DRG neurons and SCs may be exerted by oxygen at least in part, and not only by UFBs themselves. The effects of OUBs on more severe injuries such as rat sciatic nerve transection or spinal cord injury are of further interest. For future convenient clinical applications, elucidation of the effects of OUBs following oral administration will be needed.

In conclusion, OUBs accelerate nerve regeneration and functional recovery in SNC rats by promoting neurite outgrowth in DRG neurons via AKT signaling and SC proliferation via ERK1/2 and JNK/c-JUN signaling, indicating that OUBs are a hopeful treatment option for peripheral nerve injuries and diseases.

## 4. Materials and Methods

### 4.1. Preparation of UFBs Diluted in Medium and Saline

**Figure 7:**
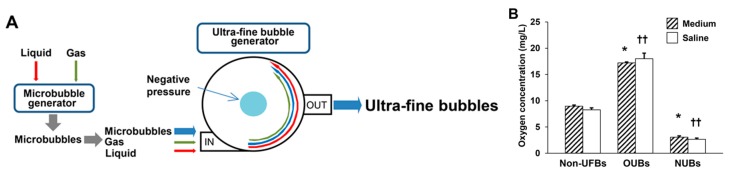
Preparation of UFBs diluted in medium and saline. (**A**) The ultra-fine bubble aerator. Fine microbubbles of gas (oxygen or nitrogen) were generated after brief sonication. Then UFBs were generated using a gas–liquid (oxygen or nitrogen; culture medium or saline) mixing system with hydrodynamic function. In this apparatus, gas was supplied at 0.1 MPa and 0.7 L/min into microbubble water for 30 min. The high-speed centrifugal force caused by the circulation separates the microbubbles into UFBs by the strong shearing force in the dispersed water; (**B**) the dissolved oxygen concentration of OUBs and NUBs diluted in medium or saline (OUB or NUB-saturated liquid, that is, OUB or NUB concentration 100%) was measured at 30 min after generation by Winkler’s method. The OUB data were analyzed by one-way ANOVA followed by Dunnett test. Graphs show mean ± SD. * *p* < 0.05 vs. non-UFB medium, †† *p* < 0.01 vs. non-UFB saline. UFBs: ultra-fine bubbles, OUBs: oxygen ultra-fine bubbles, and NUBs: nitrogen ultra-fine bubbles.

Fine microbubbles of gas were generated with brief sonication of liquid (culture medium or saline) and gas (oxygen or nitrogen) by a microbubble generator. Then, these microbubbles, liquid and gas, were placed into an ultra-fine bubble aerator (BUVITAS; Ligaric Company Limited, Osaka, Japan), which is a gas–liquid mixing system in which high-speed centrifugal force separates the microbubbles into UFBs by the strong shearing force, as previously described [2] (Figure 7A). Nitrogen was selected as a negative control, because it is abundant in the atmosphere. The UFB-saturated liquid directly generated by the ultra-fine bubble aerator was defined as 100% UFB concentration liquid. UFBs diluted in liquid were filtered immediately after generation using a 220-nm pore size cellulose acetate membrane (Corning Inc., Tewksbury, MA, USA) to avoid contamination. The UFB-saturated liquid (100% UFB concentration liquid) was gradually diluted immediately before the experiment with the same non-UFB liquid to 75, 50, and 25%, as previously described [9]. The size, concentration, and dissolved oxygen concentration of OUBs produced by these methods were stable in liquid up to day 70 at 4 °C as previously described [2]. OUBs diluted in liquid were kept at 4 °C with a tight lid, and were re-generated every 2 months. Oxygen concentration in the 100% OUB liquid was significantly elevated in both media (Non-UFBs vs. OUBs: 9.0 vs. 17.2 mg/L; *p* < 0.05) and saline (Non-UFBs vs. OUBs: 8.3 vs. 18.0 mg/L; *p* < 0.01), whereas, oxygen concentration was significantly decreased in the 100% NUB liquid in both media (Non-UFBs vs. NUBs: 9.0 vs. 3.1 mg/L; *p* < 0.05) and saline (Non-UFBs vs. NUBs: 8.3 vs. 2.7 mg/L; *p* < 0.01) (Figure 7B).

### 4.2. Animals

Animals were kept under a 12/12 h light/dark cycle (lights on 08:00–20:00 h) environment. All animals were allowed free to access to food (MF, Oriental Yeast, Osaka, Japan) and tap water. We performed all experiments in accordance with the National Institutes of Health Guide for the Care and Use of Laboratory Animals. All animal experiments were approved by the Ethics Review Committee for Animal Experimentation of Osaka University (approval number: 24-022-007, approval date: 23 August 2012). We made maximum effort to minimize the number of animals utilized and limit any suffering.

### 4.3. Surgical Procedures

**Figure 8:**
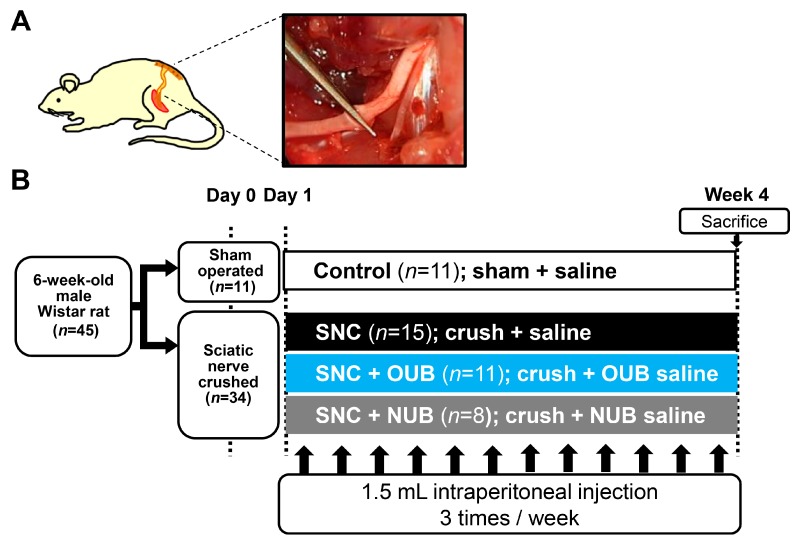
(**A**) Schematic diagram and photo of the surgical procedure. The sciatic nerve 5 mm distal from the sciatic notch was crushed three times in three different directions for 10 s per crush; (**B**) experimental protocols. SNC: sciatic nerve crush injury, OUBs: oxygen ultra-fine bubbles, and NUB: nitrogen ultra-fine bubble.

Forty-five male Wistar rats weighing 180–220 g (Charles River Laboratories, Yokohama, Japan) were used in the study. The animals underwent sciatic nerve crush as previously described [23]. Upon all experiments, animals were deeply anesthetized using subcutaneous injection which comprised of a mixture of midazolam (2 mg/kg), butorphanol (2.5 mg/kg), and medetomidine (0.15 mg/kg), and were immobilized in the prone position. The left sciatic nerve was exposed and freed from surrounding tissues from the sciatic notch to its bifurcation into the tibial and peroneal nerves. The sciatic nerve 5 mm distal from the sciatic notch was crushed three times in three different directions for 10 s per crush (Figure 8A). The fascia and the skin were closed using 4–0 nylon. In sham-operated animals, the left sciatic nerve was surgically exposed as mentioned above without any injury to the nerve. The rats were administered 1.5 mL saline, OUBs diluted in saline, or NUBs diluted in saline by intraperitoneal injection three times per week for 4 weeks beginning on the day after the operation. Rats were divided into four groups: (1) control, (sham operation + saline injections (*n* = 11)); (2) SNC, (crush injury + saline injections (*n* = 15)); (3) SNC+OUB, (crush injury + OUB-saline injections (*n* = 11)); and (4) SNC+NUB, (crush injury + NUB-saline injections (*n* = 8)) (Figure 8B).

### 4.4. SFI Analysis

To evaluate motor function, the SFI was calculated as previously described [56,57,58]. At 4 weeks after the intraneural injection, rats were made to walk across a narrow track. The hindpaws were dipped in black ink and footprints were recorded on white paper. SFI was calculated from the footprints according to the formula established. The following parameters were measured: print length (PL), which is the distance from the heel to the toe; toe spread (TS), which is the distance from the first to the fifth toes; and intermediary toe spread (ITS), which is the distance from the second to the fourth toes. PL, TS, and ITS were collected on both the normal (N) and the experimental (E) hind legs.

SFI = −38.3 × (EPL − NPL)/NPL + 109.5 × (ETS − NTS)/NTS + 13.3 × (EITS − NITS)/NITS − 8.8(1)

The SFI varies from 0 to −100: scores around 0 indicate normal nerve function and around −100 indicates complete loss of function.

### 4.5. von Frey Filament Test

To evaluate sensory function, at 2 weeks after the operation, paw withdrawal thresholds were measured to assess mechanical sensitivity with calibrated von Frey filaments (TouchTest, North Coast Medical Inc., Gilroy, CA, USA), as previously described [59,60]. Rats were placed on an elevated metal mesh and von Frey filaments were applied to the plantar surface of the hindpaw until they bent. Values were normalized to the unoperated side.

### 4.6. Electrophysiological Analysis

Electrophysiological analysis was performed at 4 weeks after the operation as previously described [23]. Rats were anesthetized and placed on a smooth table. The sciatic nerve and the tibialis anterior muscle were exposed. A pair of stimulating electrodes was noninvasively placed on the proximal side of the crush injury site. We placed a recording electrode in the tibialis anterior muscle to record the CMAP and the TL. The NCV was calculated using two different points across the crush injury site of the sciatic nerve. CMAP, TL, and NCV were detected and measured using the PowerLab device and software (version 3.8.5, AD Instruments, Bella Vista, NSW, Australia).

### 4.7. Immunostaining of Sciatic Nerves

For histological evaluation, animals were sacrificed at 4 weeks after the operation. They were anesthetized and the sciatic nerve containing the area of crush injury site was excised and embedded in 4% paraformaldehyde for 24 h at room temperature and then stored in 20% sucrose in 0.01 M phosphate-buffered saline (PBS). The tissues were embedded in the Frozen Section Media (Leica Biosystems, San Diego, CA, USA), frozen in liquid nitrogen, sectioned axially at 5 μm, and mounted on a glass slide. They were permeabilized with 100% methanol for 30 min at −20 °C. After blocking with PBS containing 0.2% Triton X and 5% bovine serum albumin (BSA; Sigma-Aldrich, St. Louis, MO, USA), they were incubated with primary antibodies against MBP (mouse; 1:1000; NE1018; Calbiochem, San Diego, CA, USA) and NF200 (rabbit; 1:1000; N4142; Sigma-Aldrich) overnight at 4 °C inside a humidified chamber, followed by incubation with the appropriate secondary antibodies including Alexa Fluor 488 goat anti-rabbit IgG (1:1000; A-11034; Molecular Probes, Eugene, OR, USA) and Alexa Fluor 568 goat anti-mouse IgG (1:1000; A-11004; Molecular Probes). The myelinated ratio was calculated as the number of both MBP- and NF200-positive axons (myelinated axons) to the number of NF-200 positive axons (total axons) using NIS Elements BR 3.00, SP3 software (Laboratory Imaging, Nikon, Tokyo, Japan).

### 4.8. Primary Culture of DRG Neurons and SCs

DRG neurons were cultured as previously described with minor modification [61]. Briefly, DRGs were obtained from Wistar rats at postnatal day 10 and dissociated by incubation with 0.25% trypsin (GIBCO/BRL Life Technologies, Grand Island, NY, USA), 0.1% collagenase (Sigma-Aldrich), and 200 U/mL DNase I (Roche Diagnostics, Mannheim, Germany). The cells were cultured on poly-l-lysine (Sigma-Aldrich)-coated 4-well chamber slides in modified Sato medium (Dulbecco’s Modified Eagle’s Medium (DMEM; GIBCO/BRL Life Technologies) containing 5 μg/mL insulin (Sigma-Aldrich), 20 nM progesterone (Sigma-Aldrich), 100 μM putrescine (Sigma-Aldrich), 30 nM sodium selenite (Sigma-Aldrich), 0.1 μg/mL l-thyroxine (Sigma-Aldrich), 0.08 μg/mL triiodo- l-thyronine (Sigma-Aldrich), and 4 mg/mL BSA) [61].

SCs were isolated and cultured as previously described [39,62]. Briefly, SCs were collected from the sciatic nerves of postnatal day 1–5 Wistar rats and cultured on poly-l-lysine-coated dishes (IWAKI, Shizuoka, Japan) in growth medium (DMEM containing 3% fetal bovine serum (Sigma-Aldrich) with 3 μM forskolin (Merck, Darmstadt, Germany) and 20 ng/mL neuregulin (R&D Systems, Minneapolis, MN, USA)). We obtained SC cultures of >99% purity using these procedures. In all experiments, cells were used between passages 3 and 8.

### 4.9. Immunocytochemistry

Immunocytochemistry was performed as previously described [23]. Briefly, DRG neurons were fixed with 4% paraformaldehyde for 30 min at room temperature. After blocking with PBS containing 0.2% Triton X and 5% BSA, the samples were incubated with primary antibodies against neuronal class III β-tubulin (Tuj1) mouse monoclonal antibody (1:1000; MMS-435P; Covance, Dedham, MA, USA) overnight at 4 °C, followed by incubation with the appropriate secondary antibodies including Alexa Fluor 488 goat anti-mouse IgG (1:1000; A-11029; Molecular Probes). The nuclei were stained with the Permafluor mounting solution containing DAPI (D532; DOJINDO, Kumamoto, Japan).

### 4.10. Neurite Outgrowth Assay

DRG neurons were cultured in OUB-diluted Sato medium (concentration 0, 25, 50, 75, and 100%) for 48 h and then immunostained with anti-TuJ1 antibody. The axonal length (the length of the longest neurite per TuJ1-positive neuron) and total neurite length per neuron were measured using a fluorescent microscope (eclipse 90i, Nikon) and a digital camera (DS-Ri1 and DS-U3, Nikon), as previously described [61]. Only neurites longer than 20 μm (about the diameter of a soma) and not in contact with other cells were measured. The axonal length and mean total neurite length were calculated from at least 30 neurons in each experiment.

### 4.11. WB

Cells were homogenized in 100 μL Kaplan buffer (150 mM NaCl, 50 mM Tris-HCl (pH 7.4), 1% NP-40, 10% glycerol, and a protease inhibitor cocktail (Roche Diagnostics)). The lysate was subject to sodium dodecyl sulphate-polyacrylamide gel electrophoresis (SDS-PAGE) and WB analysis with a standard procedure [62] using antibodies against AKT (1:1000; CST 4691; Cell Signaling Technology, Beverly, MA, USA), phospho-AKT (1:1000; CST 4056; Cell Signaling Technology), ERK1/2 (1:1000; CST 4695; Cell Signaling Technology), phospho-ERK1/2 (1:1000; CST 9101; Cell Signaling Technology), p38 (1:1000; CST 9212; Cell Signaling Technology), phospho-p38 (1:1000; CST 9215; Cell Signaling Technology), JNK (1:1000; CST 9252; Cell Signaling Technology), phospho-JNK (1:1000; CST 4668; Cell Signaling Technology), c-JUN (1:1000; CST 9165; Cell Signaling Technology), phospho-c-JUN (1:1000; CST 3270; Cell Signaling Technology), MBP (1:1000; M3821; Sigma-Aldrich), MAG (1:1000; MAB1567; Chemicon, Temecula, CA, USA), P0 (1:1000; ab31851; Abcam, Cambridge, UK), HIF1α (1:1000; CST 14179; Cell Signaling Technology), and β-actin (1:1000; CST 4970; Cell Signaling Technology). Protein expression levels were determined using the MF-ChemiBIS 3.2 imaging system (Berthold Technologies, Bad Wildbad, Germany).

### 4.12. Gene Expression Analysis

Total RNA was extracted from cells using the RNAeasy Mini Kit (Qiagen, Düsseldorf, Germany), and first-strand complementary DNA was obtained with the SuperScript III First-Strand Synthesis System (GIBCO/BRL Life Technologies) according to the manufacturer’s protocol. Quantitative real-time PCR was performed using the Step One Plus Real-Time PCR System (GIBCO/BRL Life Technologies), as previously described [9]. The expression values were normalized to hypoxanthine phosphoribosyltransferase 1 (HPRT1). PCR primers (forward and reverse, respectively) were as follows: HPRT1 (5′-CTGGTGAAAAGGACCTCTCGAA-3′ and 5′-CTGAAGTACTCATTATAGTCAAGGGCAT-3′), Krox20 (5′-TTTTTCCATCTCCGTGCCA-3′ and 5′-GAACGGCTTTCGATCAGGG-3′), Oct-6 (5′-GGCACCCTCTACGGTAATGTGT-3′ and 5′-TTGAGCAGCGGTTTGAGCT-3′), MBP (5′-CAATGGACCCGACAGGAAAC-3′ and 5′-TGGCATCTCCAGCGTGTTC-3′), MAG (5′-CGCCTTTGCCATCCTGATT-3′ and 5′-TGTGACGTTCTTTTTTCTTCTTGTCT-3′), Pmp22 (5′-GCGGAACACTTGACCCTGAA-3′ and 5′-TCATTTAAACATGTGGCCCCA-3′), P0 (5′-ACCTTCAAGGAGCGCATCC-3′ and 5′-GCCATCCTTCCAGCTAGGGT-3′), GDNF (5′-GAGAGAGGAACCGGCAAGCT-3′ and 5′-GCGACCTTTCCCTCTGGAAT-3′), PDGF-BB (5′-GTTCGGACGGTGCGAATC-3′ and 5′-GTGTGCTTAAACTTTCGGTGCTT-3′), IGF-1 (5′-AGACGGGCATTGTGGATGA-3′ and 5′-ACATCTCCAGCCTCCTCAGATC-3′), BDNF (5′-GGTATCAAAAGGCCAACTGA-3′ and 5′-GCAGCCTTCCTTGGTGTAAC-3′), NGF (5′-TCCAGGTGCATAGCGTAATG-3′ and 5′-CTCCGGTGAGTCCTGTTGAA-3′), VEGF (5′-ACGACAGAAGGGGAGCAG-3′ and 5′-AGATGTCCACCAGGGTCTCA-3′).

### 4.13. SC Differentiation Assay

For differentiation experiments, SCs were cultured in growth medium for 24 h. They were induced to differentiate by the addition of 1 mM db-cAMP (Sigma-Aldrich) to make a differentiation medium and cultured for 72 h with or without OUBs, as previously described with minor modifications [14,62,63]. The effects on the differentiation of SCs after treatment were determined with real-time PCR and WB using differentiation markers such as Krox20, Oct-6, MBP, MAG, Pmp22, and P0.

### 4.14. Cell Proliferation Assay

Cell proliferation ability was detected by cell counting as previously described with some modifications [62]. Briefly, SCs were plated at a density of 3.5 × 10^4^ cells in 35-mm plates. Cells were maintained in growth medium containing OUBs (concentration 0, 25, 50, 75, and 100%) for 1, 3, 5, and 7 days. Medium exchange was performed at days 3 and 5 using the same OUBs concentration medium respectively. Cell counting was performed with a Countess™ Automated Cell Counter (Invitrogen, Carlsbad, CA, USA).

### 4.15. BrdU Uptake Assay

Proliferation of SCs was evaluated using the BrdU uptake assay (Roche Diagnostics) according to the manufacturer’s protocol. Briefly, SCs were cultured at a density of 1.0 × 10^4^ cells/well in 96-well plates in growth medium with or without OUBs for 24 h under normoxic (20% O_2_) or hypoxic (1% O_2_) conditions as previously described with minor modification [64]. After the addition of 10 μM BrdU, cells were incubated for 2 h. The BrdU incorporation rate was measured using an automatic microplate reader at 370 nm.

### 4.16. Apoptosis Assay

Apoptosis of SCs was evaluated using Cell Death Detection ELISA^plus^ (Roche Diagnostics) according to the manufacturer’s protocol. Briefly, SCs were cultured at a density of 1.0 × 10^4^ cells/well in 96-well plates with or without OUBs for 24 h under normoxic or hypoxic conditions. The supernatant was transferred to the well of a streptavidin-coated 96-well microplate, where nucleosomes were incubated with two monoclonal antibodies: antihistone (biotin-labeled) and anti-DNA (peroxidase-conjugated). Antibodynucleosome complexes were bound to the microplate by the streptavidin. After removal of the unbound antibodies, the amount of peroxidase retained in the immunocomplex was photometrically determined with peroxidase substrate. The absorbance was measured using an automatic microplate reader at 405 nm.

### 4.17. Statistics

The data were expressed as the mean ± standard deviation (SD). Statistical analyses were performed with one-way analysis of variance (ANOVA) followed by Dunnett test or Tukey-Kramer HSD test using JMP software, version 11 (SAS Institute, Cary, NC, USA).

## Abbreviations

ANOVA	analysis of variance
BDNF	brain-derived neurotrophic factor
BrdU	5-bromo-2-deoxyuridine
BSA	bovine serum albumin
CMAP	compound muscle action potential
db-cAMP	dibutyryl cyclic AMP
DMEM	Dulbecco’s Modified Eagle’s Medium
DRG	dorsal root ganglion
ERK	extracellular signal-regulated kinase
GDNF	glial cell-derived neurotrophic factor
HIF1α	hypoxia-inducible factor 1α
HPRT1	hypoxanthine phosphoribosyltransferase 1
IGF-1	insulin-like growth factor-1
ITS	intermediary toe spread
JNK	c-Jun-N-terminal kinase
MAG	myelin-associated glycoprotein
MBP	myelin basic protein
NCV	nerve conduction velocity
NF200	neurofilament 200
NGF	nerve growth factor
ns	not significant
NUBs	nitrogen ultra-fine bubbles
Oct-6	octamer transcription factor-6
OUBs	oxygen ultra-fine bubbles
P0	protein zero
PBS	phosphate-buffered saline
PCR	polymerase chain reaction
PDGF-BB	platelet-derived growth factor-beta
PL	print length
Pmp22	peripheral myelin protein 22
PNI	peripheral nerve injury
PNS	peripheral nervous system
SCs	Schwann cells
SD	standard deviation
SFI	sciatic functional index
SNC	sciatic nerve crush injury
TL	terminal latency
TS	toe spread
Tuj1	neuronal class III β-tubulin
UFBs	ultra-fine bubbles
VEGF	vascular endothelial growth factor
WB	western blotting
